# Advances in Azole Antifungal Agents: From Structural Foundations to Resistance Challenges

**DOI:** 10.3390/jof12100728

**Published:** 2026-09-29

**Authors:** Yajing Yin, Wanrong Lu, Meng Zhao, Yueru Zhao

**Affiliations:** 1 Tianjin Key Laboratory of Food Biotechnology, College of Biotechnology and Food Science, Tianjin University of Commerce, Tianjin 300134, China; 18119902782@163.com (W.L.); 16632869596@163.com (Y.Z.); 2 Universe Gene Technology (Tianjin) Co., Ltd., Tianjin 300134, China; zhaomeng19921207@163.com

**Keywords:** azoles, structure–activity relationship, antifungal spectrum, resistance mechanisms, One Health

## Abstract

Azoles are the most widely used antifungal agents in clinical practice, with important applications in human medicine, agriculture, and veterinary science. This review systematically summarizes the structural features, classification, six-decade development history, mechanisms of action, antifungal spectra, and resistance mechanisms of azole antifungals. Based on the number of nitrogen atoms in the azole ring, these compounds are divided into imidazoles (two nitrogens) and triazoles (three nitrogens), which differ markedly in target enzyme selectivity, pharmacokinetic properties, and safety. Tetrazoles, as a new generation of drugs, exhibit lower basicity and reduced inhibition of human CYP450 enzymes, and have demonstrated improved selectivity and better safety profiles in preclinical studies; however, their clinical advantages still require further validation. Since the introduction of the first clinically used imidazole clotrimazole in 1969, azoles have evolved from topical formulations to orally or intravenously administrable triazoles across four generations, each iteration broadening the antifungal spectrum and improving safety and pharmacokinetics. Azoles act by inhibiting fungal sterol 14α-demethylase (CYP51), thereby blocking ergosterol biosynthesis and disrupting fungal cell membrane integrity. Their spectrum covers most clinically relevant fungi, but significant inter-generational differences exist. However, the widespread emergence of resistance mechanisms—including efflux pump overexpression, target enzyme alterations, biofilm formation, and other mechanisms—combined with cross-resistance risks between agricultural azole fungicides and clinical azoles, severely compromises their clinical utility. This review also discusses future directions, including novel tetrazoles, dual/multi-target inhibitors, AI-assisted drug design, and cross-sectoral resistance control within the One Health framework.

## 1. Introduction

Fungal infections have become an increasingly serious global public health threat. A systematic study incorporating data from more than 120 countries between 2010 and 2023 estimated approximately 6.5 million cases of invasive fungal infections (IFIs) annually, with about 3.8 million associated deaths, of which roughly 2.55 million (68%) are directly attributable to the fungal infection itself. Among the various IFIs, invasive aspergillosis affects over 2.11 million people each year, with a crude mortality rate of 85.2% (approximately 1.8 million deaths); invasive candidiasis affects about 1.565 million, causing 0.995 million deaths (63.6%); and cryptococcal meningitis affects 194,000, with 147,000 deaths (75.8%) [1]. The expanding population of immunocompromised individuals further exacerbates this situation [2]. Beyond invasive disease, superficial and mucosal fungal infections impose a substantial disease burden, with an estimated >1 billion people affected worldwide [3]. In the United States, the direct medical and indirect economic costs of fungal infections are estimated at $11.5 billion annually [4].

The emergence of multidrug-resistant fungal pathogens adds further complexity. Since its first description in 2009, *Candidozyma auris* (syn. *Candida auris*) has been reported in 82 countries across six continents. Its multidrug resistance, high mortality, and rapid transmissibility within healthcare settings have led the World Health Organization (WHO) to designate it as a critical public health threat [5]. Azole-cross-resistant isolates of *Aspergillus fumigatus* are now widely distributed on all continents except Antarctica [6]. In clinical settings, *Fusarium* spp., Mucorales, and *Scedosporium* spp./*Lomentospora prolificans* exhibit pan-resistance to most currently available antifungals [7,8,9]. The global spread of azole-resistant dermatophyte strains has also resulted in numerous treatment failures [10]. Resistance not only limits the clinical utility of existing drugs but also directly increases patient mortality and healthcare costs [11].

In stark contrast to the abundance of antibacterial agents, the antifungal armamentarium remains extremely limited. Currently, only four major classes are available for treating invasive fungal infections: polyenes, azoles, echinocandins, and pyrimidine analogues (5-flucytosine). Azoles, owing to their broad spectrum, oral bioavailability, and relatively acceptable toxicity profile, occupy an irreplaceable core position [12]. Since the first clinically used azole, clotrimazole, was introduced in the late 1960s, this class has undergone multiple iterations and is now extensively used not only in human medicine but also in agriculture and veterinary practice [13]. However, widespread azole use has driven increasing resistance, and cross-resistance further narrows therapeutic options [14]. Moreover, the extensive use of azole fungicides in agriculture is linked to clinical azole resistance, posing a cross-domain transmission risk under the One Health perspective [15,16]. Azole-resistant fungal infections lead to the failure of existing treatment regimens and a sharp rise in mortality, giving rise to novel non-azole antifungals. For example, olorofim is a dihydroorotate dehydrogenase inhibitor; this enzyme is a key enzyme in the pyrimidine biosynthesis pathway. Inhibition of this enzyme blocks pyrimidine synthesis, thereby preventing fungal growth and cell division, resulting in fungicidal activity. Olorofim is active against a range of hyaline moulds, including *Fusarium* species, and has completed phase III clinical trials, showing promising clinical prospects [17]. Fosmanogepix is a prodrug of manogepix and an inhibitor of the Gwt1 enzyme in the glycosylphosphatidylinositol (GPI) anchor protein biosynthesis pathway. It has high oral bioavailability and is active against various resistant filamentous fungi (including *Aspergillus* spp., *Fusarium* spp., *Scedosporium* spp., and *Rhizopus arrhizus*) and yeasts (including *Cryptococcus* and *Candida* spp.), and is currently in phase III clinical trials [17].

In summary, the global burden of fungal infections continues to rise, and the growing immunocompromised population and emergence of multidrug-resistant strains present unprecedented challenges. Given the limited number of antifungal classes, azoles remain the most widely used antifungal category. Although previous reviews have addressed individual aspects of azole antifungals, such as their medicinal chemistry [13], clinical applications [18], or resistance mechanisms in specific pathogens [19], a comprehensive and integrated synthesis that systematically links structural evolution, target interactions, species-specific resistance mechanisms, and clinical implications across major fungal pathogens has been lacking. Moreover, the widespread use of azoles in agriculture, veterinary medicine, and clinical settings has created a critical situation of cross-sectoral resistance, yet this connection has long been overlooked. This review aims to systematically address these issues by constructing a unified framework that integrates medicinal chemistry, clinical implications, and the One Health perspective, thereby providing a reference for guiding rational clinical use and promoting the development of novel antifungal agents.

## 2. Chemical Structures and Classification of Azole Antifungals

### 2.1. Core Structural Features of the Azole Ring

Azole antifungals are synthetic compounds with a nitrogen-containing five-membered aromatic heterocycle (azole ring) as the core scaffold. The azole ring is linked via its N-1 atom to a side chain that contains at least one aromatic ring. This architecture consists of two key elements: the azole ring (the “active head”) and the side chain. The nitrogen atoms of the azole ring are essential for antifungal activity. The side chain, typically containing an aromatic ring (e.g., phenyl, fluorophenyl), determines the drug’s target selectivity, pharmacokinetics, and spectrum of activity [20,21].

### 2.2. Classification by Azole Ring Type

Based on the number of nitrogen atoms in the azole ring, azoles are divided into imidazoles (two nitrogens at positions 1,3) and triazoles (three nitrogens). Triazoles are further subdivided into 1,2,4-triazole and 1,2,3-triazole isomers [22,23]. These structural differences profoundly affect target selectivity and safety. Imidazoles (e.g., clotrimazole, miconazole, ketoconazole, econazole) possess two ring nitrogens and inhibit fungal CYP51 but also exhibit relatively high affinity for human cytochrome P450 enzymes, correlating with hepatotoxicity and drug–drug interaction risks [24,25]. Triazoles (e.g., fluconazole, itraconazole, voriconazole, posaconazole) have three ring nitrogens and show significantly higher selectivity for fungal CYP51 over mammalian enzymes, thereby offering improved safety [26]. Although triazoles exhibit improved selectivity in distinguishing fungal CYP51 from host CYP450 enzymes, they still retain some inhibitory activity against human CYP3A4, CYP2C9, and CYP2C19 (particularly CYP3A4), which underlies clinically significant drug–drug interactions. This can lead to notable toxicity and adverse reactions in immunocompromised patients, especially when co-administered with other therapeutic agents [26,27].

Beyond imidazoles and triazoles, azoles also include benzimidazole and tetrazole scaffolds. Benzimidazole was the first azole compound described with antifungal activity, reported by Woolley in 1944 [28]. Tetrazoles (e.g., oteseconazole) are regarded as the next-generation direction for azole development [29].

### 2.3. Core Principles of Structure–Activity Relationship (SAR)

SAR studies are essential for understanding the link between chemical structure and biological activity and for guiding the design of new azole antifungals. The antifungal activity of azoles depends on several structural elements: (1) Coordination of azole nitrogen with heme iron: The nitrogen atom (N-3 of imidazole or N-4 of triazole/tetrazole) coordinates with the iron ion in the CYP51 heme centre, occupying the active site and blocking substrate (e.g., lanosterol) binding, thereby inhibiting CYP51 activity [13]. (2) Advantages of tetrazoles and triazoles over imidazoles: The basicity (iron-binding affinity) of the azole ring does not directly correlate with inhibitory potency but is closely linked to hepatotoxicity and drug interactions. Tetrazole has the lowest basicity (−1.1), compared with 2.3 for triazole and 6.8 for imidazole; thus, tetrazoles exhibit minimal CYP450 inhibition and the fewest related adverse effects [13,30,31]. This explains why triazoles have largely replaced imidazoles as the mainstream antifungals. Tetrazoles, as a novel structural optimization, have gained considerable attention, and the approval of oteseconazole marks a new era for azole development. (3) Side chain substituents critically influence target selectivity, spectrum, and pharmacokinetics. Side chain A is invariably a halogenated phenyl group essential for inhibitory activity, with fluorine at C-2 and C-4 providing enhanced potency [32,33]. Side chain B, similar to the heme-binding group, can be optimized to design highly effective agents with favourable pharmacokinetics [34]. Elongation of side chain B (e.g., itraconazole, posaconazole) enhances binding affinity to the hydrophobic pocket of the target enzyme, broadening the spectrum [35,36]. (4) The linker between the azole ring and side chain ranges from one to four atoms, creating specific distances that enhance potency [32]. The first atom of the linker is typically carbon and may bear substituents in topical agents (e.g., clotrimazole analogues and vinyl-imidazole derivatives). The second and third atoms are usually carbon, and their chirality is crucial for activity [36]. A hydroxyl group at C-2 of the linker provides multiple benefits: it forms indirect hydrogen bonds via water molecules with the active site, improving potency; it enhances aqueous solubility and metabolic stability; and it is required for prodrug design (e.g., fluconazole, voriconazole, ravuconazole) [37]. A methyl group at C-3 increases activity against moulds; its chiral configuration places side chain B antiperiplanar to side chain A and fills the C-13 binding pocket of lanosterol [38].

### 2.4. Lipophilicity and Membrane Permeability

The ability of azole antifungals to cross the fungal plasma membrane is a critical determinant of their bioavailability and efficacy. Key physicochemical properties (log*P* and pKa) are major factors governing the rate of passive diffusion across membranes [39,40]. The pKa determines the fraction of drug in the un-ionized form at physiological pH, and this un-ionized form is the predominant species for transmembrane diffusion. A higher log*P* indicates greater lipophilicity, which facilitates penetration through the fungal cell membrane; however, excessively high lipophilicity reduces aqueous solubility, leading to poor oral absorption. These differences also underlie the distinct pharmacokinetic behaviors among different azole drugs. For example, although clotrimazole is highly lipophilic (log*P* ≈ 5), its extremely low aqueous solubility and extensive first-pass metabolism result in poor oral bioavailability, and it is therefore used mainly as a topical agent [41,42,43]. In contrast, fluconazole, despite its relatively low lipophilicity (log*P* ≈ −0.44), exhibits high oral bioavailability and wide tissue distribution, including penetration into the cerebrospinal fluid, owing to its high aqueous solubility and low plasma protein binding [44,45,46]. Itraconazole, on the other hand, is highly lipophilic (log*P* ≈ 5.98) but has poor aqueous solubility, and its oral bioavailability is markedly influenced by food and gastric acidity, which in turn affect tissue distribution and clinical efficacy [47,48,49]. This highlights the importance of balancing lipophilicity and aqueous solubility in azole drug design and in the selection of appropriate agents for specific clinical situations.

## 3. Development History and Generational Evolution

The generational classification adopted in this review is based largely on structural similarity, major breakthroughs, and chronological sequence. However, this classification scheme is not uniformly standardized across the literature, and we acknowledge that different authors may apply different classification criteria.

### 3.1. Early Discoveries (1944–1968)

**Figure 1:**
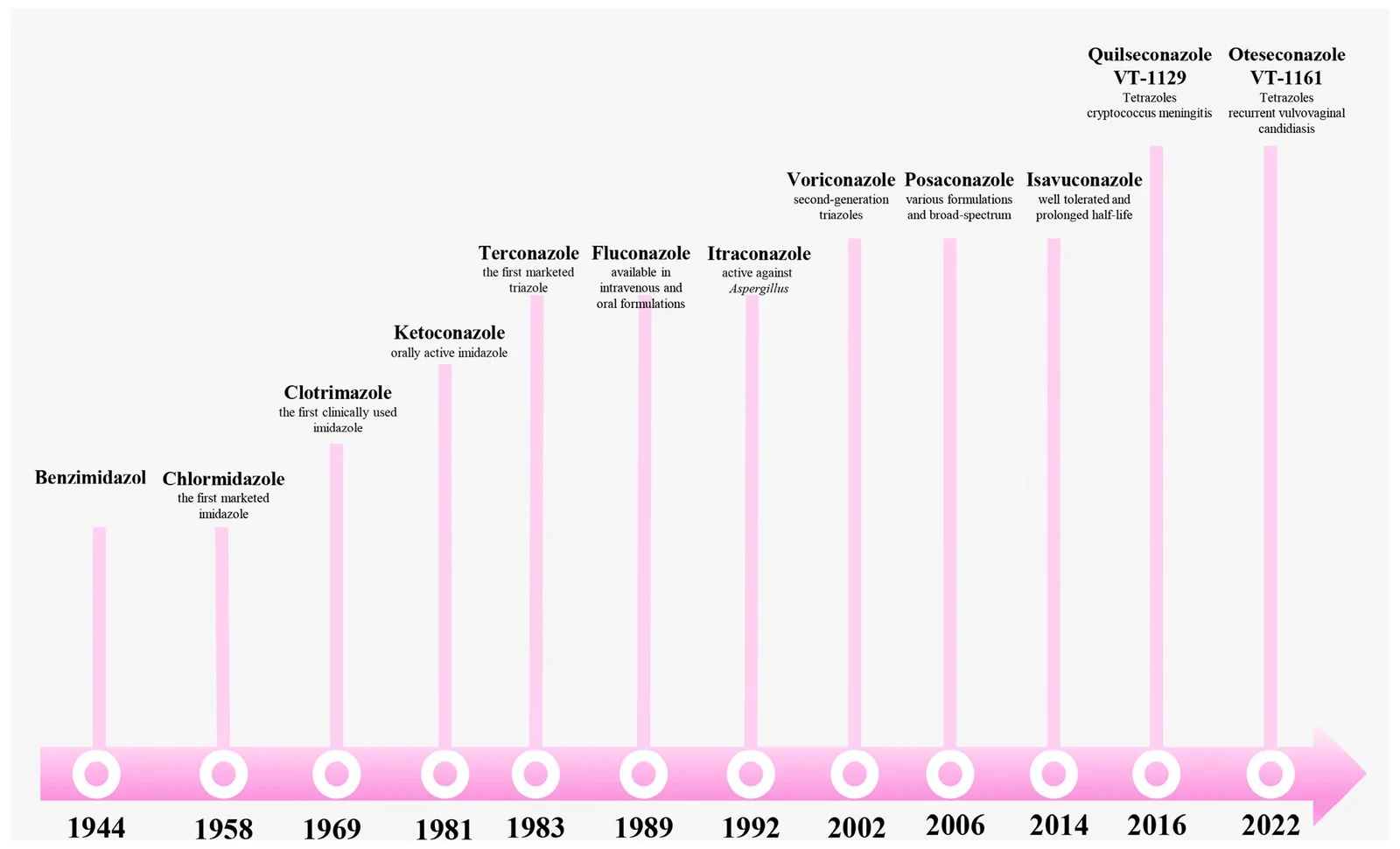
Timeline of azole antifungal development (1944–2022). Key milestones in the discovery and approval of azole antifungals are shown chronologically. The timeline highlights the evolution from the first azole with antifungal activity (benzimidazole, 1944) to the most recently approved tetrazole (oteseconazole, 2022). Important milestones include the introduction of the first marketed azole (chlormidazole, 1958), the first clinically used imidazole (clotrimazole, 1969), the first orally active azole (ketoconazole, 1981), the first triazole (terconazole, 1983), and the approval of the first tetrazole (oteseconazole, 2022).

**Figure 2:**
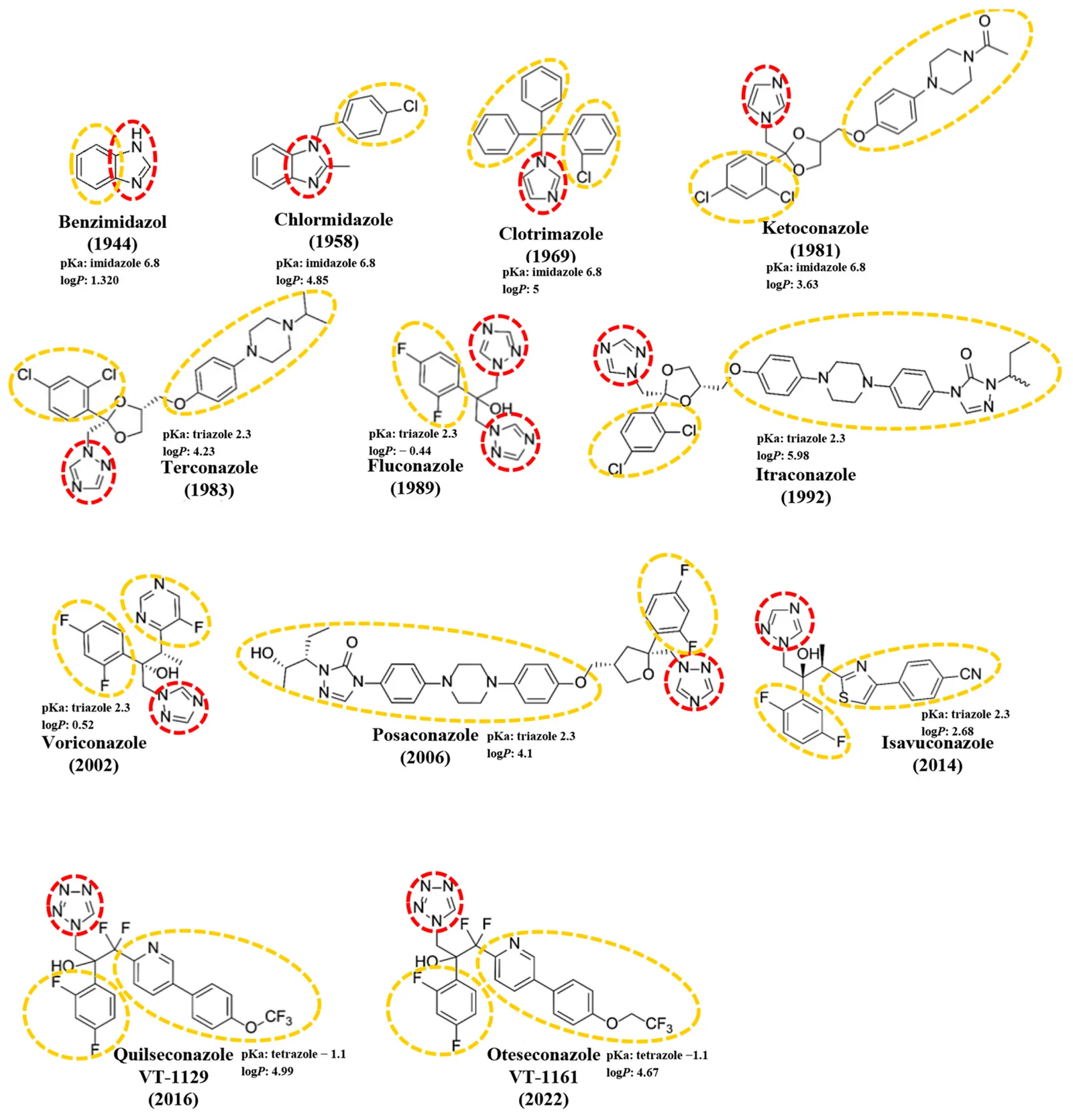
Comparison of core structural scaffolds of the four major azole classes. This figure illustrates the evolution of the azole ring scaffold from the earliest imidazoles to the latest tetrazole derivatives, using representative azole drugs from each generation. pKa: conjugate acid dissociation constant of the azole ring nitrogen; log*P*: Octanol–water partition coefficient. The pKa and log*P* values for each azole drug are listed below the corresponding structure; all values are predicted [13], and predicted values may vary between different models. Red dashed boxes: the azole ring (imidazole/triazole/tetrazole), which can coordinate to the CYP51 heme iron. Yellow dashed boxes: side chain A (the halogenated phenyl ring responsible for π–π stacking) and side chain B (which occupies the CYP51 substrate entry channel).

The antifungal activity of azoles was first described in 1944, when Woolley reported that benzimidazole possessed antifungal properties [28] (Figure 1 and Figure 2). Subsequent researchers identified other azole structures with antifungal activity, such as phenethylimidazole [50] and substituted benzimidazoles [51], but none led to a marketed drug. In 1958, chlormidazole, developed by Chemie Grünenthal, became the first azole antifungal to reach the market, sparking extensive research into azoles for antifungal therapy. Today, about 40 azole-containing drugs and drug candidates exist [41,52,53], and the field remains highly active, owing to the urgent need for antifungal treatments.

### 3.2. First-Generation Azoles: Imidazoles (1969–1970s)

In 1969, Bayer AG introduced clotrimazole (Figure 1 and Figure 2), the first imidazole antifungal to enter clinical use. Shortly thereafter, Janssen Pharmaceutica launched miconazole (also 1969), followed by econazole in 1974. These three drugs were initially marketed as topical antifungal agents, establishing azoles as indispensable tools for treating fungal infections [41]. Subsequent similar compounds, such as isoconazole, with comparable physicochemical properties and imidazole-containing structures, were also used for superficial infections (e.g., dermatophytoses and vaginal candidiasis) and thus are grouped as first-generation imidazoles [42,43]. However, these agents have poor oral absorption and low bioavailability, restricting them largely to topical use. Although miconazole could be administered intravenously, rapid infusion caused adverse effects including nausea, fever, and cardiotoxicity due to the Cremophor^®^ EL formulation (BASF, Ludwigshafen, Germany) required for solubilization [54].

### 3.3. Second-Generation Azoles: Orally Active Imidazoles (1980s)

The approval of ketoconazole in 1981 was a milestone (Figure 1 and Figure 2), designated as a second-generation imidazole. It introduced a heterocyclic dioxolane functional group and a long-tail substituent, while retaining the imidazole moiety. Unlike previous imidazoles, ketoconazole was the first orally active, broad-spectrum azole for systemic mycoses [55]. However, its clinical utility was soon limited by significant hepatotoxicity (due to low selectivity for fungal versus mammalian CYP450), poor bioavailability, extensive drug interactions, poor central nervous system penetration, and anti-androgenic endocrine effects. These shortcomings prompted a shift towards safer triazoles, and ketoconazole was eventually removed from the FDA’s list of approved drugs for systemic mycoses in 2013 [56].

### 3.4. Third-Generation Azoles: Triazoles (1980s–1990s)

The advent of triazoles represented another major leap. Replacing the imidazole moiety with a triazole ring conferred superior solubility, broader spectrum, and higher selectivity and safety due to greater affinity for the fungal target enzyme [57]. Terconazole, a novel triazole ketal synthesized by Janssen in 1983 (Figure 1 and Figure 2), was the first triazole antifungal to be approved, but it was restricted to topical use and active against dermatophytes, pathogenic yeasts, and *Candida* spp. [58]. Fluconazole, itraconazole, and fosfluconazole belong to the triazole class. Fluconazole, developed by Pfizer and approved in 1989 [59], was optimized through structural modifications to achieve enhanced selectivity for fungal CYP over mammalian CYP, a broader spectrum, greater metabolic stability, lower protein binding, improved bioavailability, better water solubility (allowing for intravenous use), good CNS penetration, and fewer side effects [44,45,46]. To allow for even higher intravenous bolus doses, a phosphate ester prodrug (fosfluconazole) was developed and approved in 2003; it is converted to fluconazole by alkaline phosphatase, enabling more rapid intravenous administration [60]. Fluconazole’s antifungal spectrum covers the vast majority of *Candida* and *Cryptococcus* species, and it was approved in 1990 as a single-dose oral treatment for vaginal candidiasis [61]. Candidemia is the most common form of invasive candidiasis and is associated with high mortality, particularly in critically ill and immunocompromised patients. Current clinical guidelines generally recommend echinocandins as initial empirical therapy, especially in critically ill patients, those with prior azole exposure, or neutropenic patients. Fluconazole serves as an alternative for initial therapy only in non-critically ill patients without recent azole exposure who are infected with fluconazole-susceptible strains. In addition, after pathogen identification and susceptibility testing results are available, fluconazole can be used for targeted therapy. It may also serve as step-down therapy in clinically stable patients who have responded well to initial echinocandin therapy and are infected with a fluconazole-susceptible strain. Therefore, the clinical use of fluconazole depends on the fungal species, susceptibility profile, prior azole exposure, underlying disease, host characteristics, and clinical stability. Its safety profile led to rapid replacement of ketoconazole for many indications, but resistance has emerged over recent decades. *Nakaseomyces glabrata* (formerly *Candida glabrata*) exhibits reduced susceptibility to fluconazole, and resistance may be associated with treatment failure, particularly in patients with prior azole exposure [62]. *C. parapsilosis* is generally susceptible to fluconazole, but resistance rates have been increasing globally and warrant vigilance. *Pichia kudriavzevii* (formerly *C. krusei*) is intrinsically resistant to fluconazole; fluconazole should not be used for empirical therapy, and therefore echinocandins are the preferred initial therapeutic agents. Thus, the relationship between antifungal resistance, treatment failure, and mortality varies by species and should not be generalized to all fungal pathogens. Itraconazole, developed by Janssen, followed soon after [41]. It has a broader spectrum than fluconazole, including activity against *Aspergillus* spp., a second-line agent for the treatment of aspergillosis [63,64], and lower hepatotoxicity than ketoconazole [52]. However, its oral bioavailability is more variable and affected by gastric acidity and food intake [47,48,49]. Itraconazole exhibits substantial interpatient variability in pharmacokinetics and concentration-dependent toxicity; therefore, routine therapeutic drug monitoring (TDM) is recommended [65]. Despite improved safety and spectrum over ketoconazole, triazoles still suffer from drug–drug interactions, toxicity, resistance, and pharmacokinetic limitations.

### 3.5. Newer Triazoles (2000s to Present)

Since 2000, newer triazoles have been introduced to broaden the spectrum, enhance safety, improve pharmacokinetics, overcome resistance, and even achieve fungicidal activity against some species. Voriconazole, the first low-molecular-weight, water-soluble second-generation triazole, was approved by Pfizer in 2002 (Figure 1 and Figure 2). It is a fluconazole derivative in which a fluoropyrimidine ring replaces the second triazole group [66], dramatically expanding the spectrum. Voriconazole shows superior activity against *Aspergillus* spp., now first-line for invasive aspergillosis [67]. It also has activity against refractory *Fusarium* and *Scedosporium* infections [68]. In addition, it is >30-fold more potent than fluconazole against *Candida albicans*, 20-fold more potent against *N. glabrata*, and retains activity against fluconazole-resistant or less-susceptible *N. glabrata* and *P. kudriavzevii*, a recommended agent for systemic candidiasis and esophageal candidiasis [67]. Adverse effects include skin rash, elevated transaminases [69], encephalopathy, and hallucinations [70,71,72]. Voriconazole exhibits pronounced interpatient pharmacokinetic variability and has a narrow therapeutic window, with toxicity being concentration-dependent; therefore, routine TDM is recommended to maintain plasma concentrations within the therapeutic range and minimize toxicity [65].

Posaconazole, an itraconazole analogue approved in 2006 [73,74], plays a central role in the prophylaxis and treatment of invasive fungal diseases (IFDs), especially in immunocompromised patients. Its broad spectrum and multiple formulations (oral suspension, delayed-release tablets, intravenous solution) make it versatile [75,76]. Posaconazole has a broad antifungal spectrum, including activity against many clinically relevant yeasts and moulds, with additional activity against Mucorales; however, activity against some difficult-to-treat moulds is species- and isolate-dependent. It has been clinically used as a second-line treatment for mucormycosis, and is also effective in central nervous system (CNS) fungal infections and cryptococcal meningitis [77]. It also shows synergy with echinocandins (e.g., caspofungin) and amphotericin B [78,79]. Overall tolerability is good, even with long-term use; most adverse effects are mild (nausea, vomiting) [77,80], although hepatotoxicity has been reported [81]. Challenges include drug–drug interactions and variable oral absorption [75,76], especially in patients on antiretroviral therapy [82]. Another notable limitation is the high cost [83] and concentration-dependent toxicity; in such cases, TDM is recommended, particularly for oral formulations [65,84]. However, heterogeneity in study designs complicates direct comparisons.

Isavuconazole is a novel broad-spectrum triazole, valuable for IFI treatment, particularly in hematological malignancy patients. Isavuconazonium sulfate was approved by the US FDA in 2015 for the treatment of invasive aspergillosis and invasive mucormycosis, and it shows comparable efficacy to that of voriconazole and liposomal amphotericin B for these indications [85,86]. It is generally well-tolerated, with fewer adverse effects and treatment discontinuations than other agents [87]. Although up to 29% of patients develop elevated liver enzymes, discontinuation is rarely required [88], and it is suitable for complex leukemia regimens [89]. Isavuconazole exhibits relatively stable pharmacokinetics, with TDM required only in specific high-risk situations, and its prolonged half-life (~184 h) permits once-daily dosing, improving adherence and outpatient management [90]. It also shows prophylactic potential in high-risk hematological populations, though further large-scale studies are needed [91,92]. The water-soluble prodrug isavuconazonium sulfate (developed by Basilea and Astellas) is rapidly converted to isavuconazole by plasma esterases, eliminating the need for cyclodextrin solubilization and reducing nephrotoxicity risk [93].

Ravuconazole, a fluconazole derivative, exhibits a broader spectrum and greater potency than fluconazole, with activity against fluconazole- and itraconazole-resistant infections [13]. It was discontinued from phase II trials for aspergillosis and candidiasis in August 2007, but its prodrug fosravuconazole bis(L-lysine) was developed by Bristol-Myers Squibb and first approved in Japan in 2018 as NAILIN^®^ 100 mg capsules for onychomycosis [94].

Efinaconazole, a novel topical triazole for mild-to-moderate onychomycosis, received its first global approval in Canada in October 2013; it offers a better safety profile than oral therapies [95].

Other novel antifungals are advancing rapidly. Opelconazole is in phase III trials for invasive pulmonary aspergillosis (OPERA-T, NCT05238116) [96]; its unique nebulised administration delivers high local lung concentrations while minimizing systemic exposure, thereby reducing typical azole toxicities and drug interactions [97]. Opelconazole has broad in vitro activity against *Aspergillus* spp., *Candida* spp., *Trichophyton rubrum*, *C. gattii*, *C. neoformans*, *Penicillium chrysogenum*, and *R. oryzae* [98]. Preclinical and clinical data indicate good tolerability with mild adverse effects and no significant pulmonary function changes; ongoing development focuses on treatment and prophylaxis of pulmonary aspergillosis in immunocompromised or chronic lung disease patients [97]. Albaconazole, containing a quinazolinone scaffold, is orally active and broad-spectrum [99]. A phase II trial for distal subungual onychomycosis showed high cure rates and good tolerability, superior to placebo and an alternative to terbinafine and itraconazole [100].

### 3.6. Fourth-Generation Azoles—Tetrazoles

A major issue with azoles is inhibition of human CYP3A4, leading to complex drug–drug interactions [42]. Although replacing imidazole with triazole improved selectivity, the problem persisted. Tetrazoles have emerged as a promising new subclass, representing a trend towards even higher selectivity and safety. Viamet Pharmaceuticals, starting from voriconazole, replaced the high-affinity metal-coordinating triazole with a low-affinity tetrazole and compensated for reduced activity through side-chain modifications, yielding fourth-generation compounds with superior selectivity, potency, and favourable pharmacokinetic and physicochemical properties [30,101]. Quilseconazole (VT-1129) has entered phase I trials for cryptococcal meningitis; in murine models it significantly prolonged survival compared with fluconazole, and received FDA Fast Track and orphan drug designations in 2016 (Figure 1 and Figure 2) [102]. Oteseconazole (VT-1161), an oral agent, was approved by the FDA in 2022 as Vivjoa for recurrent vulvovaginal candidiasis (RVVC) [29]; it also shows activity against fluconazole-resistant *N. glabrata* and *P. kudriavzevii* [103]. However, its long half-life poses embryofetal toxicity risks, so use during pregnancy or lactation is not recommended. VT-1598 has the broadest spectrum among tetrazoles [104,105], with notable anti-coccidioidomycosis activity, advancing to preclinical development in the USA [106]; it also exhibits good activity against multidrug-resistant fungi such as *C. auris* [107].

Tetrazoles have achieved significant improvements in selectivity and safety, as their azole ring exhibits the lowest basicity and minimal inhibition of human CYP450 enzymes [13,30,31]. However, these structural advantages do not necessarily ensure that tetrazoles can overcome established mechanisms of azole resistance. Because tetrazoles and triazoles share the same binding site in CYP51, target-site mutations such as Y132F and K143R do reduce the binding affinity and activity of tetrazoles; nevertheless, the impact on tetrazoles (e.g., VT-1161 and VT-1129) is generally smaller than that observed with short-tailed triazoles such as fluconazole and voriconazole [108]. Notably, the Y132F single mutation and the Y132F&K143R double mutation have a minimal effect on VT-1598 MICs (showing no appreciable change or only a 2-fold increase), indicating that VT-1598, as a tetrazole, exhibits better tolerance to target-site mutations [109]. In addition, studies have shown that the susceptibility of VT-1161 can be reduced by overexpression of ABC and MFS efflux pumps, although the extent to which tetrazoles are affected by efflux-mediated resistance requires further investigation [110]. Thus, while tetrazoles offer important safety advantages, they should not be regarded as a universal panacea for triazole-resistant infections.

### 3.7. Clinical Pharmacology and Safety of Azoles

In addition to the chemical structural and mechanistic considerations mentioned above, the clinical use of azole antifungals is also constrained by their adverse effect profiles, drug–drug interactions, and pharmacokinetic limitations. Voriconazole, a trifluorinated azole, is associated with fluoride excess and the development of periostitis/exostosis, whereas other fluorinated triazoles (fluconazole and posaconazole) do not increase the risk of hyperfluorosis or its complications. Therefore, voriconazole is indeed considered unique among triazoles in being associated with this toxicity, possibly related to the fluorine atom on its pyrimidine ring [111]. Notably, tetrazoles (VT-1129, VT-1161, and VT-1598) contain a greater number of fluorine atoms (7, 7, and 4, respectively), although these are not located on a pyrimidine ring. The safety of their long-term use, including the potential risk of elevated plasma fluoride concentrations and the occurrence of periostitis, remains to be further verified. Inhibition of steroid metabolism by azoles (e.g., posaconazole and itraconazole) can lead to pseudohyperaldosteronism [112], whereas ketoconazole has been formally approved for the treatment of Cushing’s disease because of its potent inhibition of adrenal steroidogenesis [113]. Azoles, whether imidazoles or triazoles, are metabolized by the hepatic cytochrome P450 enzyme system and are also inhibitors of these enzymes. This inhibitory effect gives rise to clinically significant drug–drug interactions, leading to markedly elevated plasma concentrations of co-administered drugs metabolized by hepatic cytochrome P450 enzymes, thereby increasing or prolonging their therapeutic effects and adverse reactions, and potentially causing severe toxicity. This is an important reason limiting the clinical use of azole antifungals [108]. Pharmacokinetic limitations also exist; due to high protein binding, many azoles (e.g., itraconazole) penetrate poorly into urine, cerebrospinal fluid, and vitreous humor [114]. Several systemic azoles have demonstrated embryo–fetal toxicity or teratogenic effects in animal studies, and their use during pregnancy is generally restricted or requires careful assessment of the potential risks and benefits. These limitations underscore the need for therapeutic drug monitoring and the development of next-generation azoles with improved safety profiles and better tissue distribution characteristics.

## 4. Mechanism of Action

### 4.1. Target: Sterol 14α-Demethylase (CYP51/ERG11)

The target of azole antifungals is sterol 14α-demethylase, encoded by the *ERG11* gene (*CYP51* in *Aspergillus*), a member of the cytochrome P450 superfamily. CYP51 is a key enzyme in ergosterol biosynthesis, catalyzing the oxidative removal of the 14α-methyl group from sterols to generate C14-demethylated sterols. This is a rate-limiting step in ergosterol biosynthesis. The substrate for CYP51 differs among fungal species. In *Neurospora crassa* [115], *A. fumigatus* [116], *F. graminearum* [117] and *C. neoformans* [118], lanosterol is converted by sterol C24-methyltransferase (ERG6) to eburicol, which then serves as the CYP51 substrate to produce 4,4-dimethylergosta-8,14,24(28)-trien-3β-ol, subsequently processed by ERG24, the sterol C4-demethylase complex, and ERG2 to ergosterol. In *Saccharomyces cerevisiae* and the pathogenic yeast *C. albicans* [119], lanosterol itself is the substrate for CYP51, producing 4,4-dimethylcholesta-8,14,24(25)-trien-3β-ol, which is then processed by ERG24, the C4-demethylase complex, and ERG6 to ergosterol. Ergosterol is an essential component of the fungal cell membrane, critical for fluidity, permeability, and integrity.

### 4.2. Molecular Mechanism of Action

The antifungal action of azoles involves several steps. First, the azole nitrogen coordinates with the heme iron in CYP51, anchoring the drug in the active site [120,121]. Second, the side chain interacts with amino acid residues in the active site via hydrogen bonds, π-π stacking, and hydrophobic interactions [122], enhancing binding affinity and determining selectivity for different fungal CYP51 orthologues. Third, drug binding irreversibly inhibits the oxidative removal of the 14α-methyl group, blocking ergosterol biosynthesis [57]. The resulting dual effect—depletion of ergosterol and accumulation of toxic 14α-methylated sterol intermediates—disrupts membrane structure and function [116,119], leading to growth inhibition (primarily fungistatic for most azoles).

### 4.3. Fungistatic and Fungicidal Activity

Whether the antifungal activity of azoles acts primarily through cytostatic or cytotoxic effects depends mainly on the working concentration and the target fungal species. Against yeasts (e.g., *Candida* spp.), azoles generally exhibit fungistatic activity. One study showed that even at high concentrations (up to 10 µg/mL), itraconazole and voriconazole were unable to kill *C. albicans* and only inhibited its growth. In contrast, against molds (e.g., *Aspergillus* spp.), azoles can exert fungicidal activity. The same study found that exposing *A. fumigatus* conidia to various concentrations (1.25 to 10 µg/mL) of itraconazole and voriconazole resulted in cell death; this cytotoxic effect was enhanced with prolonged time and increasing concentration [123]. Subsequent studies also confirmed that the minimum fungicidal concentration (MFC) of voriconazole against *Aspergillus* spp. ranges from 1 to 2 µg/mL [124]. This distinction is of great significance for clinical treatment, especially for immunocompromised patients. In patients with impaired immune function (e.g., organ transplant recipients, those undergoing cancer chemotherapy, HIV-infected individuals, etc.), their own immune system struggles to eliminate pathogens that have been inhibited by the drug. Therefore, fungicidal activity may be more advantageous in such patients, helping to more thoroughly eradicate the pathogen [125].

## 5. Antifungal Spectrum

### 5.1. Commonly Susceptible Fungi

Azoles cover the vast majority of clinically relevant human fungal pathogens [126,127]. *Candida* spp. are among the most important targets, with *C. albicans* generally susceptible to most azoles [128]. *C. neoformans* primarily causes subacute and chronic infections of the meninges, with high mortality. Fluconazole plays a major role in the consolidation and maintenance phases of cryptococcal meningitis treatment, serving to clear residual infection and prevent relapse. For the induction phase, an amphotericin B-based combination regimen is recommended. Fluconazole resistance and heteroresistance in *C. neoformans* warrant particular attention [129]. Heteroresistance refers to the presence of a resistant subpopulation within an otherwise susceptible clonal population; this subpopulation can transiently expand under drug selection pressure, leading to treatment failure or relapse. This phenotype is usually unstable but may complicate clinical management. Furthermore, validated clinical resistance breakpoints for azoles (including fluconazole) against *C. neoformans* have not yet been established. Available epidemiological cutoff values (ECOFF/ECV) are intended to distinguish wild-type from non-wild-type isolates and should not be interpreted as clinical resistance breakpoints [130,131]. *Aspergillus* spp. susceptibility varies by agent: fluconazole is essentially inactive, while voriconazole and posaconazole show excellent activity [132,133]. Dermatophytes (e.g., *Trichophyton*, *Microsporum*) are susceptible to many topical azoles (clotrimazole, luliconazole, itraconazole, miconazole, efinaconazole), but fluconazole has poor activity and high resistance rates [41,43,95,134,135]. Endemic fungal pathogens (e.g., *Histoplasma*, *Coccidioides*) are susceptible to itraconazole and voriconazole [136,137].

### 5.2. Comparative Spectra Across Generations

First-generation imidazoles (miconazole, clotrimazole) have broad spectra but are limited to topical use (Table 1) [41,43]. Ketoconazole, the first oral imidazole, is effective against *Candida* and *Cryptococcus* but weak against *Aspergillus* spp., and its hepatotoxicity limits systemic use [55,56]. Fluconazole is active against *Candida* spp., *C. neoformans*, *Histoplasma*, *Blastomyces*, and *Coccidioides*, but lacks mould activity and is used clinically for local and systemic candidiasis and systemic cryptococcosis [138,139]. Itraconazole has a broader spectrum including yeasts and *Aspergillus*. It is clinically used for the treatment of oral and esophageal candidiasis and as a second-line agent for aspergillosis [140,141]. However, both are poorly active against emerging pathogens such as *Scedosporium*, *Fusarium*, and Mucorales [142,143]. Voriconazole and posaconazole address these gaps, showing in vitro activity against *Fusarium*, *Scedosporium*, *C. neoformans*, and Mucorales [18,144]. Voriconazole is first-line for invasive aspergillosis due to its superior activity against *A. fumigatus*, but it may cause adverse reactions such as visual disturbances, hepatotoxicity, and drug–drug interactions. For *Scedosporium* species, the clinical efficacy of voriconazole varies by species. For example, voriconazole is commonly used to treat *S. apiospermum* infections but is usually ineffective against *L. prolificans*. However, voriconazole has no clinically relevant activity against Mucorales and should not be used to treat mucormycosis. Posaconazole is clinically used for the prevention of invasive aspergillosis or candidiasis, and is also used for the treatment of mucormycosis, especially as salvage therapy or step-down therapy in patients who are intolerant to amphotericin B or in whom treatment has failed, but its in vitro activity against *Fusarium* species is variable depending on the species [145]. Isavuconazole has a spectrum similar to voriconazole and posaconazole. It is a recommended agent for invasive aspergillosis and mucormycosis. Compared with voriconazole, isavuconazole has a better safety profile and fewer drug–drug interactions. These differences should be fully considered when selecting treatment for individual patients with invasive aspergillosis. The clinical evidence for isavuconazole in mucormycosis is mainly based on single-arm trials and case series which is weaker than that for invasive aspergillosis [85,86]. Ravuconazole, a fluconazole derivative, has broader and more potent activity, including against fluconazole-/itraconazole-resistant strains [13,18]; its prodrug is used for onychomycosis [94]. Efinaconazole is for mild-to-moderate onychomycosis [95]. Tetrazoles represent the next generation: quilseconazole for cryptococcal meningitis [102]; oteseconazole for RVVC [29], active against fluconazole-resistant *N. glabrata* and *P. kudriavzevii* [103]; and VT-1598, with the broadest tetrazole spectrum, shows significant activity against *Coccidioides* and has entered preclinical development in the United States. It also shows good inhibitory activity against multiple drug-resistant fungi, such as *C. auris* [105,106,107].

### 5.3. Intrinsic and Acquired Resistance

Some species are intrinsically resistant to certain azoles. *P. kudriavzevii* is intrinsically resistant to fluconazole due to structural differences in CYP51 that prevent effective binding [146]. Acquired resistance arises under drug selection pressure; prolonged or repeated azole exposure selects for resistant subpopulations. This makes the spectrum concept more complex, as different isolates of the same species may show varying susceptibility. Global surveillance indicates that while *C. albicans* fluconazole resistance remains low overall, the proportion of non-wild-type isolates is gradually increasing [147,148]. In contrast, *C. auris* shows markedly reduced fluconazole susceptibility; monitoring by the USA Centers for Disease Control and Prevention (CDC) Antimicrobial Resistance Laboratory Network found that 95% of 8033 clinical isolates were fluconazole-resistant [149].

## 6. Current Status and Mechanisms of Resistance

### 6.1. The Severity of Resistance

Azole resistance is one of the most serious challenges in antifungal therapy. Fluconazole resistance rates are rising, and multidrug-resistant *C. auris* has spread globally [149,150]. In *Aspergillus*, azole resistance in *A. fumigatus* is increasing in several regions [6,151,152]. Not only does resistance diminish the efficacy of individual drugs, but cross-resistance further restricts clinical choices. It directly leads to higher treatment failure rates, prolonged hospitalization, and increased mortality [151]. Moreover, azole resistance is not confined to clinical settings. In modern agriculture, azole fungicides (such as tebuconazole, propiconazole, and epoxiconazole) are extensively used to control fungal diseases in cereals, fruits, vegetables, and cash crops. Agricultural azole fungicides share structural similarities with clinical azoles and target the same CYP51 enzyme, making them significant environmental drivers of clinical resistance selection [15,16]. The emergence of azole-resistant *A. fumigatus* genotypes TR34/L98H and TR46/Y121F/T289A has been definitively linked to agricultural fungicide exposure, and these mutations have been detected in soil, plant debris, and air samples from multiple agricultural regions across Europe, Asia, and the Americas [15,153,154]. Patients who are initially susceptible to azoles can acquire resistant infections after inhaling environmental resistant spores, leading to cross-resistance, which has been confirmed in several regions with high fungicide usage [6]. Although the association between agricultural azole use and clinical azole resistance is increasingly recognized, the strength of the evidence varies across regions and fungal species. In *A. fumigatus*, this link is most firmly established, with TR34/L98H and TR46/Y121F/T289A mutations strongly associated with environmental azole exposure. However, for *Candida* species, the evidence is more circumstantial, and the relative contribution of agricultural versus clinical azole use to resistance selection remains difficult to quantify. *Candida* species are primarily human commensals and are not major plant pathogens. Therefore, the direct selection pressure exerted by agricultural azole fungicides on *Candida* is indeed less clear than that on *A. fumigatus*. However, in vitro cross-resistance and indirect environmental evidence suggest that this route may still contribute to clinical resistance. For example, the agricultural triazole tebuconazole can rapidly select for *C. auris* variants with fluconazole cross-resistance and echinocandin tolerance under laboratory conditions [155]. A study isolated fluconazole-resistant *C. tropicalis* from orchard environments in Taiwan. Its genotype was highly related to resistant strains causing human candidemia, and fluconazole susceptibility was positively correlated with susceptibility to three agricultural azole fungicides. This suggests that agricultural environments are a potential source of azole-resistant *C. tropicalis* [156]. However, epidemiological evidence for the contribution of agricultural azoles to clinical azole resistance remains insufficient. Key limitations include the lack of standardized environmental surveillance, the difficulty of distinguishing between cross-resistance selection and co-selection, and the confounding effects of widespread clinical azole use. Future research should prioritize longitudinal surveillance integrating clinical, agricultural, and environmental data to better define the causal pathways and inform evidence-based mitigation strategies [157,158]. Meanwhile, the One Health framework should be applied cautiously at the genus level, and the environmental drivers of resistance should be assessed separately for different fungal pathogens rather than generalized across genera.

### 6.2. Major Resistance Mechanisms

Fungal infections cause a broad spectrum of diseases, from superficial to life-threatening invasive forms, with high mortality in critically ill and immunocompromised patients [2]. In 2022, the WHO Fungal Priority Pathogens List (FPPL) designated *C. albicans*, *C. auris*, and *A. fumigatus* as critical-priority pathogens [11]. Azole resistance has emerged as a serious public health concern driven by multiple factors. This section summarizes the molecular mechanisms of azole resistance in these major fungal pathogens and compares species-specific resistance strategies [159].

Before delving into the molecular mechanisms of azole resistance, it is necessary to clarify several biologically distinct but often conflated concepts. Stable antifungal resistance refers to a heritable reduction in drug susceptibility in fungi, usually caused by mechanisms such as target gene mutations or efflux pump overexpression. This resistance phenotype remains stably inherited even in the absence of drug selection pressure and is an important cause of clinical treatment failure. Tolerance refers to the ability of cells or a subpopulation to survive transient drug exposure without a change in minimum inhibitory concentration (MIC). Tolerant cells usually regain their original susceptibility after drug removal and do not involve heritable resistance mechanisms, but may lead to pathogen persistence during treatment. Heteroresistance refers to the presence of a resistant subpopulation within an otherwise susceptible fungal population; this subpopulation can transiently expand under drug selection pressure, leading to treatment failure or relapse. The heteroresistance phenotype is usually unstable but has important clinical implications and can only be detected by special methods such as population analysis profiles. Adaptive responses refer to transient, non-heritable changes in gene expression or physiology that occur in fungi under drug pressure and enable cell survival. Such responses do not alter the MIC but can help fungi survive the drug exposure period and may create conditions for the subsequent emergence of stable resistance mutations. The above concepts differ significantly in biological nature, genetic basis, detection methods, and clinical significance. The molecular resistance mechanisms discussed below mostly involve gene mutations, deletions, and overexpression, which fall under stable resistance. Clarification of these concepts provides a clearer framework for clinical resistance surveillance and therapeutic strategies.

(1) Efflux pump overexpression. This is the major mechanism for high-level azole resistance in clinical *Candida* isolates, particularly *C. albicans* [160,161]. Two families are involved: ATP-binding cassette (ABC) transporters and major facilitator superfamily (MFS) transporters. Among ABC transporters, Cdr1p and Cdr2p (encoded by *CDR1* and *CDR2*) are the most important; they consist of two transmembrane domains and two nucleotide-binding domains, and utilize ATP hydrolysis to pump azoles out [161,162,163,164,165,166,167]. Overexpression of both *CDR1* and *CDR2* synergistically reduces azole susceptibility [168,169,170]. In the MFS, Mdr1p is a fluconazole-specific transporter (also reported for ketoconazole and voriconazole in some studies, but specificity for other azoles unconfirmed) [161,171]. Efflux pump expression is regulated by transcription factors: Tac1p activates *CDR1* and *CDR2*, and gain-of-function mutations cause constitutive overexpression [161,172,173]; Mrr1p regulates *MDR1* [171]. In *N. glabrata*, *CgPDR1* mutations upregulate *CgCDR1* and *CgCDR2*, causing cross-resistance to all azoles [174,175,176]. In *C. auris*, *CDR1* deletion abrogates triazole resistance, indicating that CDR1 is more critical than MDR1 [177]. Chromosomal aneuploidy can also amplify resistance genes: *C. albicans* can form an isochromosome of chromosome 5 left arm, doubling the copy number of dozens of genes including *ERG11* and *TAC1*; similar events occur in *C. auris* and *N. glabrata* [174,178,179,180]. In *C. albicans*, aneuploidy also contributes to heteroresistance, where a small subpopulation within a clonal population exhibits enhanced drug tolerance, leading to treatment failure [181,182]; similar phenomena occur in *N. glabrata* and *C. parapsilosis* [183,184]. Standard broth microdilution susceptibility testing may need to be supplemented with population analysis profiles or careful examination of disk diffusion data to detect heteroresistant subclones [185].

In azole-resistant clinical isolates of *A. fumigatus*, ABC transporters such as cdr1B (abcB/abcC/abcG1) and atrF are constitutively overexpressed [186,187]. In addition, several putative multidrug resistance (MDR) transporters, including Mdr1, Mdr2, Mdr3, and Mdr4, have been implicated in triazole resistance, although their specific contributions require further genetic validation [186,188].

(2) Target alteration and overexpression. Target gene mutations and overexpression constitute the major resistance mechanisms in *A. fumigatus*. Mutations in the *cyp51A* gene (such as G54, G138, M220, and G448) reduce drug-binding affinity, enabling the fungus to tolerate azole therapy. These mutations are widely distributed among clinical isolates and are associated with cross-resistance to multiple azoles, directly contributing to treatment failure [189,190,191,192]. Tandem repeats (TRs) in the *cyp51A* promoter region lead to overexpression of the target enzyme, often in combination with coding-region mutations such as TR34/L98H and TR46/Y121F/T289A. These TR-associated genotypes predominate among environmental isolates and confer cross-resistance to multiple azoles [193,194]. The transcription factors SrbA and AtrR bind to the TR34 sequence, inducing *cyp51A* overexpression and potentially exacerbating triazole resistance [194,195]. Mutations in HapE can also lead to *cyp51A* overexpression and pan-azole resistance, as has been demonstrated in clinical isolates [196].

In *C. albicans*, over 140 amino acid substitutions have been reported, concentrated in three “hot-spot” regions: amino acids 105–165, 266–287, and 405–488 [197,198]. Among these, Y132F and K143R are the most significant for fluconazole resistance; they reduce azole binding affinity or interfere with substrate entry, allowing for continued ergosterol production even in the presence of drugs. Different substitutions confer varying resistance levels, and double substitutions are more effective than single ones [199,200,201]. The Y132F substitution has also been found in resistant isolates of *C. tropicalis*, *C. parapsilosis*, and *C. auris* [202,203,204]. In *C. parapsilosis*, other *ERG11* mutations (e.g., G458S, K134R, K128N) have been observed, but Y132F remains dominant globally [205]. In *N. glabrata*, mutations at Y141 and S410 are associated with azole resistance [206]. In *C. auris*, nearly all fluconazole-resistant clinical isolates harbor three mutations: F126L (South Africa), Y132F (South Asia), or K143R (South America), which increase MICs only for fluconazole and voriconazole, not for itraconazole or posaconazole [207]. Notably, the lipophilic side chains of itraconazole and posaconazole may interact with additional residues along the ligand access channel, preserving activity despite Erg11p substitutions [207].

Upc2p, a zinc-cluster transcription factor, contributes to *ERG11* overexpression, especially in *C. albicans* and *C. tropicalis* [160,208]. The C-terminal domain of Upc2p senses cellular ergosterol; in a sterol-rich environment, it remains repressed in the cytosol, but upon ergosterol depletion, ligand-free Upc2p becomes active, translocated to the nucleus, and activates transcription. Gain-of-function mutations in the C-terminal domain (e.g., G648D, A643V) cause constitutive activation [209,210,211]. However, not all isolates with *ERG11* overexpression carry *UPC2* mutations, indicating other mechanisms also contribute [208].

(3) Biofilm formation. Biofilm formation is an important mechanism of drug resistance in fungi, particularly in *Candida* species. Biofilms are structured communities of fungal cells attached to surfaces and encased in extracellular polymeric substances; they limit drug penetration, protect against host immunity, and confer significant antifungal resistance [212,213]. *C. albicans* biofilm cells are more resistant to fluconazole than planktonic cells. All non-albicans *Candida* species can form biofilms, but to varying degrees: *N. glabrata* forms weaker biofilms than *C. parapsilosis* and *C. tropicalis*; *C. parapsilosis* biofilms are thinner, with high carbohydrate and low protein content; *C. tropicalis* biofilms consist of dense yeast networks, with low carbohydrate and protein content; and *N. glabrata* biofilms are more cohesive, with higher levels of both [214,215]. *C. auris* isolates form large aggregates due to incomplete budding, contributing to drug resistance in tissues and the environment. The molecular mechanisms of biofilm-mediated azole resistance require further study [216,217,218,219,220].

(4) ERG3 mutations and bypass of toxic sterol accumulation

*ERG3* encodes sterol C-5 desaturase. When azoles inhibit Erg11p, lanosterol is converted to the toxic intermediate 14α-methylergosta-8,24-dien-3,6-diol, causing growth arrest. Loss-of-function mutations in *ERG3* prevent accumulation of this toxic sterol, allowing for the accumulation of alternative non-toxic 14α-methyl sterols and conferring cross-resistance [180]. For example, loss of ERG3 function in *C. albicans* causes azole resistance [221,222]. In *N. glabrata*, ERG3 can mutate after exposure to echinocandins, leading to cross-resistance to fluconazole [223]. In *C. auris*, ERG3 mutations also occur after exposure to echinocandins [224]. In *C. parapsilosis*, ERG3 mutations have been found in strains resistant to both azoles and echinocandins [225]. Some studies suggest that strains lacking ergosterol biosynthetic enzymes may activate stress signaling pathways in response to antifungals [226,227]. Additionally, Efg1p negatively regulates *ERG3* expression, and *EFG1* mutations increase antifungal susceptibility in *C. albicans* [228,229].

The antifungal mechanism of azoles involves inhibition of the cytochrome P450 enzyme lanosterol 14α-demethylase (CYP51/Erg11), thereby blocking ergosterol synthesis and leading to the accumulation of toxic sterols produced by Δ-5,6-desaturase (Erg3) [180]. The relative contribution of these two mechanisms is mainly determined by drug concentration. At low concentrations, ergosterol depletion is usually the predominant mechanism. Loss of ergosterol disrupts membrane fluidity, permeability, and the function of membrane-bound enzymes, primarily resulting in growth inhibition. At high concentrations, the accumulation of toxic 14α-methylated sterols becomes the major factor causing growth arrest and, in some cases, fungicidal activity [116,230,231]. In addition, the predominant mechanism varies among different azole drugs. For example, in both *S. cerevisiae* and *C. albicans*, fluconazole inhibits vacuolar acidification, and exogenous ergosterol supplementation restores cell growth, indicating that ergosterol depletion is the dominant mechanism of fluconazole antifungal activity [232]. The concentration threshold for the shift from ergosterol depletion to toxic sterol accumulation varies by species and drug and has not been precisely defined.

(5) Stress response mechanisms. Hsp90 is a highly conserved molecular chaperone and a global regulator of stress response signaling networks, responsible for regulating the conformation and function of diverse client proteins, including key regulatory proteins in cell signaling [221]. Hsp90 potentiates both basal azole tolerance and the emergence of resistance in multiple fungi, including *C. albicans*. Two main mechanisms have been described: (i) Hsp90 depletion reduces biofilm growth and maturation, decreasing biofilm resistance to azoles [233]; and (ii) Hsp90 stabilizes client proteins critical for fungal growth and survival under stress, including calcineurin, Mkc1, and Cek1 MAP kinases [221,222,234]. For example, *ERG3* mutations that prevent toxic intermediate accumulation are calcineurin-dependent [221]. The regulation of client proteins by Hsp90 plays a broader role in resistance than Hsp90 alone [221,222]. Hsp90 has also been implicated in azole tolerance in *C. auris* [221,235].

(6) Reduced azole uptake and decreased intracellular accumulation. Although azole resistance is predominantly attributed to efflux pump overexpression, target alterations, and biofilm formation, increasing evidence indicates that reduced azole uptake leads to decreased intracellular drug accumulation and thereby promotes resistance. It is generally believed that azoles enter fungal cells solely by passive diffusion. However, recent studies have identified several nonspecific permeases involved in azole uptake in *S. cerevisiae* [236]. For example, the myo-inositol transporter Itr1 mediates the uptake of fluconazole, ketoconazole, and clotrimazole; the fatty acid transporter Fat1 participates in ketoconazole uptake; and the purine-cytosine permease Fcy2 is involved in fluconazole uptake. In addition, the iron transporters Ftr1 and Fet3 have been found to exert indirect effects on the efficacy of fluconazole and clotrimazole [237]. Downregulation or loss-of-function mutations in these permeases can reduce intracellular drug accumulation, thereby decreasing azole susceptibility. For instance, deletion of the myo-inositol transporter ScItr1 rendered yeast cells more resistant to some of the most commonly used azoles [237]. In *C. albicans*, fluconazole represses the expression of CaITR1 [238], suggesting a possible link between this transporter and azole uptake. All fungal pathogens express homologs of the ScItr1 transporter, but their functions have not yet been characterized. Reduced azole uptake is an important and often overlooked determinant of azole resistance, and its relative contribution to overall resistance remains incompletely defined and warrants further investigation.

(7) ROS accumulation and oxidative stress. Reactive oxygen species (ROS) are unstable, highly reactive molecules produced during cellular metabolism. Studies have reported that ROS generation occurs in both yeasts (e.g., *Candida*, *Nakaseomyces*, *Cryptococcus*) and molds (e.g., *Aspergillus*) after exposure to azole drugs [239]. ROS may damage cellular macromolecules, participate in programmed cell death (PCD), and enhance antifungal activity. Pretreatment with an ROS scavenger (N-acetylcysteine) has been shown to antagonize the killing effect of itraconazole against *C. albicans* [240]. However, it remains unclear whether these “pro-death” ROS are a direct result of the antifungal mechanism of action or a secondary product of drug-induced PCD. In addition, ROS may act as a signal to trigger adaptive stress responses, increasing the probability of antifungal resistance mutations and promoting the emergence of resistance [241]. Therefore, the interplay between ROS-mediated cell killing and stress response-mediated adaptation is an important factor influencing azole susceptibility and resistance.

While efflux pump overexpression is the predominant mechanism in *C. albicans* clinical isolates, the relative importance of different resistance mechanisms varies markedly across species. In *C. auris*, CDR1 plays a more critical role than MDR1, and clade-specific ERG11 mutations (F126L, Y132F, K143R) are nearly universal among fluconazole-resistant isolates [207]. In *A. fumigatus*, resistance is primarily driven by cyp51A mutations, particularly TR34/L98H and TR46/Y121F/T289A, which are often linked to environmental azole exposure [193,194]. These species-specific differences have important clinical implications for empirical therapy selection and resistance surveillance.

### 6.3. From Structure to Resistance and Clinical Outcome

**Figure 3:**
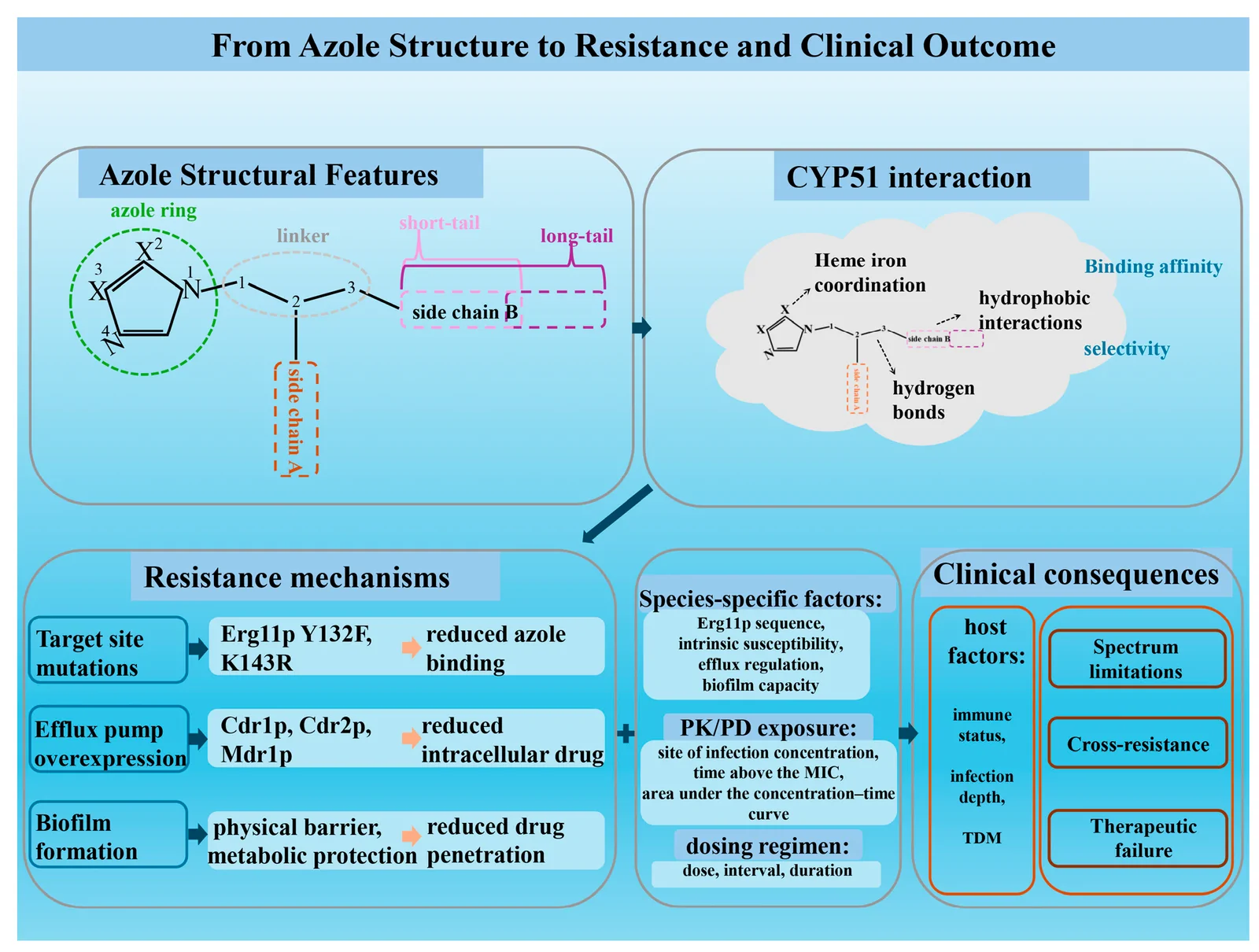
Integrative framework linking azole structural features, CYP51 interaction, resistance mechanisms, and clinical consequences. The figure illustrates how structural variations (e.g., azole ring, side chains) determine CYP51 binding affinity and selectivity. Target site mutations (e.g., Y132F, K143R in Erg11p) differentially affect azole binding. Efflux pump overexpression (Cdr1p, Cdr2p, Mdr1p) reduces intracellular drug accumulation, while biofilm formation provides a physical barrier and metabolic protection. The resulting clinical outcome is further modulated by species-specific pathogen characteristics, dosing regimen, host factors and by the pharmacokinetic/pharmacodynamic (PK/PD) exposure of the drug. These integrated factors collectively influence spectrum limitations, cross-resistance, and therapeutic failure.

The structural features of azoles, particularly the azole ring type and side chain modifications, are core determinants of target affinity and selectivity. These structural differences dictate the binding mode of the drug to the target enzyme CYP51 and ultimately influence the activity spectrum and resistance risk. The azole ring type forms the foundation of CYP51 binding affinity and selectivity (Figure 3). Imidazoles (two nitrogen atoms) exhibit relatively high affinity for both human and fungal CYP51 but have poor selectivity, which is often associated with a higher risk of hepatotoxicity and drug–drug interactions [24,25]. Triazoles (three nitrogen atoms) show significantly higher selectivity for fungal CYP51 over the human enzyme. For example, fluconazole exhibits 540-fold higher selectivity for *C. albicans* CYP51 (CaCYP51) than for human CYP51 (HsCYP51) [26,242]. Tetrazoles (four nitrogen atoms) represent the latest direction in structural optimization, offering even greater selectivity. Oteseconazole (VT-1161), for instance, potently inhibits CaCYP51 but shows no inhibition of human CYP51, with a selectivity exceeding 2000-fold [13,30,31,243]. Side chain modifications are critical in determining antifungal activity, selectivity, and the ability to overcome resistance. Short-tail azoles (e.g., fluconazole, voriconazole) possess shorter side chains and rely primarily on the coordination bond between the azole nitrogen and heme iron, as well as limited hydrophobic interactions, for target binding. This makes them highly susceptible to target site mutations that lead to resistance. For example, common *ERG11* mutations in *C. auris* (F126L, Y132F, K143R) increase the MICs of fluconazole and voriconazole by 8- to 16-fold, but have a smaller effect on itraconazole and posaconazole [207]. Long-tail azoles (e.g., itraconazole, posaconazole) contain extended lipophilic side chains that engage in extensive hydrophobic interactions with additional amino acid residues in the CYP51 active site, such as those in the ligand access channel. This multi-modal binding allows long-tail azoles to maintain appreciable binding affinity even when active-site mutations occur, rendering them relatively less susceptible to target-site resistance. For instance, in *A. fumigatus*, the Y121F and T289A mutation combination is required for high-level acquired resistance to fluconazole and voriconazole. The impact of individual *CYP51A* alterations on different triazoles varies according to the specific resistance mechanism. Therefore, the potential use of an alternative azole in azole-resistant *A. fumigatus* should be guided by antifungal susceptibility testing and the resistance profile rather than assumed on the basis of structural class alone [133]. Members of the Mucorales are intrinsically resistant to short-tail azoles (fluconazole and voriconazole), whereas their susceptibility to medium-tail (e.g., isavuconazole) and long-tail azoles (e.g., posaconazole) varies by species and can be exploited for salvage therapy [244].

The physicochemical properties of azoles, such as molecular size and lipophilicity, influence their capacity to serve as substrates for efflux pumps, thereby leading to differential tolerance of different generation azoles to efflux-mediated resistance mechanisms. Overexpression of ABC transporters (Cdr1p, Cdr2p, CgCdr1p) elevates the MICs of all tested azoles (both short-tail and long-tail), indicating that both classes are substrates of ABC transporters. In contrast, expression of the MFS transporter Mdr1p does not affect the MICs of posaconazole, itraconazole, or isavuconazole, suggesting that MFS transporters have greater substrate selectivity, primarily transporting short-tail azoles with limited impact on long-tail derivatives. Because ABC transporters can recognize multiple azoles, their overexpression generally leads to cross-resistance to all azoles. By contrast, *MDR1* overexpression primarily confers specific resistance to fluconazole, with a narrower cross-resistance profile [245].

Although the above structural features provide a theoretical basis for understanding azole antifungal resistance, it is important to note that structural features alone cannot predict clinical efficacy. Multiple additional factors must be considered, including fungal species, specific resistance mutations, combinations of resistance mechanisms, the MIC of the drug, pharmacokinetic/pharmacodynamic (PK/PD) exposure, site of infection, and host characteristics such as immune status and underlying disease. As mentioned above, the impact of specific target mutations on azole susceptibility varies depending on conditions such as efflux pump overexpression. In addition, in vitro susceptibility does not equate to clinical activity. Therefore, structural features are closely related to resistance mechanisms but cannot directly predict clinical outcome. Clinical decisions should integrate susceptibility testing, PK/PD parameters, site of infection, and host factors to optimize antifungal therapy.

### 6.4. Differences in the Rate of Azole Resistance Development

To better illustrate the dynamics of azole resistance, we summarized the rates of resistance development among major clinically used azoles. In *C. albicans*, fluconazole resistance rates remain relatively low in most regions, but the proportion of resistant isolates has shown a gradual upward trend over the past two decades [147]. *C. auris* exhibits markedly reduced susceptibility to fluconazole; monitoring by the US CDC found that 95% of isolates were resistant to fluconazole [149]. In contrast, *A. fumigatus* is intrinsically not susceptible to fluconazole. In addition, acquired resistance to mould-active triazoles may develop through *CYP51A*-mediated mechanisms, including environmentally selected TR34/L98H and TR46/Y121F/T289A genotypes [133]. Short-tail azoles (e.g., fluconazole, voriconazole) have shorter side chains and are susceptible to target mutations, leading to resistance. Long-tail azoles (e.g., itraconazole, posaconazole) have extended side chains that can bind more residues in the CYP51 ligand access channel, making them relatively less susceptible to target mutations. For example, common *ERG11* mutations in *C. auris* (F126L, Y132F, K143R) only increase the MIC of fluconazole and voriconazole (8- to 16-fold) and have less effect on itraconazole and posaconazole [207]. Despite these insights, longitudinal surveillance data remain fragmented and insufficient for effective comparison across different azoles and fungal species. Therefore, we recommend that future studies prioritize systematic long-term monitoring of azole resistance trends, ideally integrating clinical, agricultural, and environmental data within a One Health framework. Such efforts are essential to guide clinical treatment, optimize antifungal stewardship, and predict the emergence of resistance to new azoles.

## 7. Conclusions and Future Perspectives

Azole antifungals remain the cornerstone of antifungal therapy. Since the introduction of clotrimazole in 1969, azoles have evolved over more than half a century, from topical imidazoles to orally/intravenously available triazoles across four generations, each iteration broadening the spectrum, improving safety, and optimizing pharmacokinetics. Triazoles, owing to their higher selectivity for fungal CYP51, broader spectrum, and better safety, have become the dominant azole class. Agents such as fluconazole, itraconazole, voriconazole, posaconazole, and isavuconazole play irreplaceable roles in prophylaxis and the treatment of invasive fungal infections. However, resistance—driven by target mutations, efflux pump overexpression, biofilm formation, and other mechanisms—poses the greatest challenge. The link between agricultural azole use and clinical resistance further elevates this to a systemic One Health issue involving human, animal, and environmental health.

Future research should focus on the following aspects: (1) Structural optimization and development of novel tetrazoles to minimize off-target effects on human CYP enzymes. Preclinical stage: VT-1598 has the broadest antifungal spectrum among tetrazoles and also shows good inhibitory activity against *Coccidioides* species and *C. auris* [104,105,106,107]; early clinical stage: quilseconazole (VT-1129) has entered phase I clinical trials for cryptococcal meningitis and received Fast Track and orphan drug designations in the United States [102]; clinically validated example: oteseconazole (VT-1161) is an oral antifungal agent approved by the US Food and Drug Administration (FDA) for the treatment of recurrent vulvovaginal candidiasis (RVVC) [103]. (2) Dual-target inhibitors to reduce the risk of resistance from single-target mutations. Preclinical stage: dual-target inhibitors of fungal CYP51and HDAC (histone deacetylase) have been designed and show potent antifungal activity against azole-resistant *Candida* [246]. Fluconazole–cyclooxygenase (COX) inhibitor hybrids retain activity against mutant strains lacking the CYP51 target [247]. The anticancer drug osimertinib enhances azole efficacy and overcomes resistance by co-targeting Pdr5 (an efflux pump) and Vma1 (a vacuolar ATPase) [248]. (3) AI-assisted drug design to accelerate discovery of novel lead compounds. Preclinical stage: an AI generative platform named MolDiffusion was developed for small-molecule design; approximately 50% of the candidate molecules showed measurable activity in vitro, and two of them exhibited significant in vivo efficacy in a murine candidiasis model [249]. Another study used the Deep Learning–Quantitative Structure–Activity Relationship Empirical Screening (DL-QSARES) platform to identify four antifungal peptides (AFPs) with MIC values below 10 µg/mL against four tested fungal pathogens, and AFP-13 showed excellent therapeutic efficacy in animal models [250]. (4) Cross-sectoral resistance control within a One Health framework. Speculative: The use of agricultural triazole fungicides is an important driver of environmental resistance. Regulatory, agricultural, and health sectors need to strengthen communication and collaboration. This includes restricting the use of agricultural azole fungicides that are structurally similar to clinical drugs and have documented cross-resistance, and establishing a cross-sectoral resistance surveillance system that integrates agricultural fungicide use data, environmental resistance genes, and clinical resistance monitoring to develop control strategies [158]. (5) Combination therapies and novel delivery systems to improve efficacy and reduce toxicity. Preclinical stage: the combination of the calcineurin inhibitor tacrolimus with posaconazole increased in vitro efficacy against azole-resistant *A. fumigatus* by 4-fold [251]. Efflux pump inhibitors (EPIs) increase intracellular azole concentrations by inhibiting efflux pump activity, thereby reducing the in vitro MIC of azoles [252]. Non-antifungal drugs such as amiodarone and minocycline also show in vitro synergistic antifungal activity when combined with azoles [253,254]. However, most combination therapies remain limited to in vitro studies and animal models, and clinical trial data are still lacking. In addition, lipid-based nanoparticle delivery systems have been shown to overcome fluconazole resistance in *Candida* by enhancing drug penetration and bypassing efflux pump-mediated resistance [255]. Through multidisciplinary collaboration and continued innovation, it is possible to slow the spread of resistance, expand the clinical utility of azoles, and ultimately improve outcomes for patients with fungal infections.

## Figures and Tables

**Table 1:** Antifungal spectrum of major azoles.

Azoles	*Candida* spp.	*Aspergillus* spp.	*Cryptococcus* spp.	Mucorales	*Fusarium* spp.	*Scedosporium* spp.	*Histoplasma capsulatum*
Chlormidazole	+ (almost *C. albicans*)	?	+ (*C. gattii*: –)	?	?	?	?
Clotrimazole	+++ (almost *C. albicans*)	+	+	?	+	?	+
Ketoconazole	+++ (almost *C. albicans*)	–	+	–	–	–	+++
Terconazole	+++ (almost *C. albicans*)	+	?	?	?	?	?
Fluconazole	+++ (*C. auris*: –; *P. kudriavzevii:* –; some *N. glabrata*: –)	–	+++	–	–	–	±
Itraconazole	+++ (some *P. kudriavzevii*: –; some *N. glabrata*: –)	+++ (*A. lentulus*: –)	++	–	–	± (*L. prolificans*: –)	+++
Voriconazole	+++ (*C. auris*: –; some *N. glabrata*: –)	+++ (*A. lentulus*: –)	++	–	+++	+++ (*L. prolificans*: –)	++
Posaconazole	+++	+++	++	+++	± (*F. solani:* –)	++	++
Isavuconazole	++	+++	++	+++	±	++	++
Quilseconazole	+	?	+? (early clinical)	?	?	?	?
Oteseconazole	+++	–	+	+	–	?	+

Activity categories: +++, established clinical activity; ++, salvage/alternative use; +?, investigational activity; +, in vitro activity; –, generally inactive; ±, variable or species-dependent activity; ?, insufficient evidence. Footnotes: ^1^ This table summarizes in vitro susceptibility data and may not directly predict clinical efficacy. Activity should be interpreted in the context of specific clinical breakpoints, pharmacokinetic/pharmacodynamic parameters, and host factors. ^2^ For drugs with multiple entries (e.g., fluconazole against *Candida* spp.), the predominant activity is shown; exceptions are noted in parentheses. ^3^ Activity against *Fusarium* spp. and *Scedosporium* spp. may vary between species; refer to specific susceptibility data for detailed interpretation. ^4^ The tetrazoles (quilseconazole and oteseconazole) are newer agents; activity against certain fungi remains to be fully characterized.

## Data Availability

No new data were created or analyzed in this study. Data sharing is not applicable to this article.
